# Production of a Bacteriocin Like Protein PEG 446 from *Clostridium tyrobutyricum* NRRL B-67062

**DOI:** 10.1007/s12602-023-10211-1

**Published:** 2024-01-22

**Authors:** Siqing Liu, Shao-Yeh Lu, Maulik Patel, Nasib Qureshi, Christopher Dunlap, Eric Hoecker, Christopher D. Skory

**Affiliations:** 1 U.S. Department of Agriculture, Agricultural Research Service, National Center for Agricultural Utilization Research, Renewable Product Technology Research Unit, Peoria, IL 61604 USA; 2 Oak Ridge Institute for Science and Education (ORISE), Oak Ridge, TN 37830 USA; 3 U.S. Department of Agriculture, Agricultural Research Service, National Center for Agricultural Utilization Research, Bioenergy Research Unit, Peoria, IL 61604 USA; 4 U.S. Department of Agriculture, Agricultural Research Service, National Center for Agricultural Utilization Research, Crop Bioprotection Research Unit, Peoria, IL 61604 USA

**Keywords:** Anaerobic, Genome, Clostridia, *Bacillus subtilis*, Antibacterial, Recombinant protein

## Abstract

*Clostridium tyrobutyricum* strain NRRL B-67062 was previously isolated from an ethanol production facility and shown to produce high yields of butyric acid. In addition, the cell-free supernatant of the fermentation broth from NRRL B-67062 contained antibacterial activity against certain Gram-positive bacteria. To determine the source of this antibacterial activity, we report the genome and genome mining of this strain. The complete genome of NRRL B-67062 showed one circular chromosome of 3,242,608 nucleotides, 3114 predicted coding sequences, 79 RNA genes, and a G+C content of 31.0%. Analyses of the genome data for genes potentially associated with antimicrobial features were sought after by using BAGEL-4 and anti-SMASH databases. Among the leads, a polypeptide of 66 amino acids (PEG 446) contains the DUF4177 domain, which is an uncharacterized highly conserved domain (pfam13783). The cloning and expression of the *peg446* gene in *Escherichia coli* and *Bacillus subtilis* confirmed the antibacterial property against *Lactococcus lactis* LM 0230, *Limosilactobacillus fermentum* 0315-25, and *Listeria innocua* NRRL B-33088 by gel overlay and well diffusion assays. Molecular modeling suggested that PEG 446 contains one alpha-helix and three anti-parallel short beta-sheets. These results will aid further functional studies and facilitate simultaneously fermentative production of both butyric acid and a putative bacteriocin from agricultural waste and lignocellulosic biomass materials.

**Supplementary Information:**

The online version contains supplementary material available at 10.1007/s12602-023-10211-1.

## Introduction

Butyric acid is an important chemical feedstock that is used for industrial polymers, cosmetic and pharmaceutical components, and as important food additives [1]. It is currently produced chemically through petroleum refining and biologically via bacterial anaerobic fermentation. Among the microbes capable of producing butyric acid, the *Clostridium tyrobutyricum* species is thus far the best butyric acid producer. Production of butyric acid has been reported from renewable biomass residues including sweet sorghum stalks, beet molasses, paper mill sludge hydrolysates, corn steep liquor, cane molasses and oilseed rape straw, soybean hull, corn fiber, wheat straw, rice straw, and sugarcane bagasse [2–8]. These studies demonstrate that *C. tyrobutyricum* strains have great potential for sustainable production of butyric acid from agricultural sugars [9]. Most industrial microbes converting biomass to biofuels exhibit a preference for glucose over xylose (known as carbon catabolite repression (CCR)), and metabolic engineering strategies using *C. tyrobutyricum* to improve xylose utilization efficiency were reported [10, 11]. Researchers have turned to genome sequencing, with a view to assist the precise design of metabolic engineering strategies of this important species. So far, genome sequences of strains of *C. tyrobutyricum* including UC7086, ATCC 25755, KCTC 5378, L319, DIVETGP, and CCTCC W428 have been reported [11–16] in addition to the draft genome of 12 different strains of *C. tyrobutyricum* [17].

In this study, we sequenced the genome of *C. tyrobutyricum* NRRL B-67062 searching for new value-added co-products in parallel with increased butyric acid fermentation. During our investigations to enhance butyric acid production utilizing the NRRL B-67062 [5], we discovered that the filtered spent broth from anaerobic fermentation using strain B-67062 exhibited distinct antibacterial properties, not associated with butyric acid, against Gram-positive organisms. Our aim here is to analyze the genome of *C. tyrobutyricum* NRRL B-67062 to understand the unique antibacterial properties. We have carried out bioinformatics analyses searching for bacteriocin-like proteins. Among the leads, one potential uncharacterized protein encoded by *peg* 446 was analyzed and reported here. To the best of our knowledge, the present study is the first to report the novel antibacterial property of PEG 446.

## Materials and Methods

### Antibacterial Activity Assay

Anaerobic butyric acid fermentation using *C. tyrobutyricum* NRRL B-67062 in 100 mL Reinforced Clostridial Medium (RCM, Difco, Thermo Fisher Scientific, USA) was carried out from 5 mL overnight culture of a single colony on MRS plate (Difco, Thermo Fisher Scientific) at 37 °C for 24 h as described previously [5]. The cell-free supernatant (CFS) was obtained from fermentation broth after centrifugation at 8000 × *g* followed by membrane filtration (0.2 *µ*M Nalgene polyethersulfone (PES) filter, Thermo Fisher Scientific, USA). The pH of the CFS was adjusted to 6.5 using 1 M sodium hydroxide and filtered through a 3 kDa membrane (Amicon filter, Millipore, USA) by centrifugation. The sample-labeled C. tyro 3K top contained retentate with molecules retained on top of a 3 kDa membrane, and the flowthrough sample was labeled 3K bottom. Additional membrane filtration by loading 3K top sample into a 10 kDa membrane (Amicon filter, Millipore, USA) resulted in 10K top (containing molecules above 10 kDa) and 10K bottom (containing molecules between 3 and 10 kDa) samples. These fractions were used for Biolector screening assay against a model Gram-positive target *Lactococcus lactis* LM 0230 cells that we have used previously as described [18].

### Genome Sequencing

*C. tyrobutyricum* strain NRRL B-67062 from glycerol stock was streaked out on MRS plate and was incubated at 37 °C in an anaerobic chamber (Coy Laboratory, USA) for 24–48 h until colonies became visible. The plate was maintained in an anaerobic jar at 4 °C in a refrigerator. A single colony of *C. tyrobutyricum* NRRL B-67062 was grown overnight in strict anaerobic condition in 5 mL RCM [5, 6]. Total genomic DNA was extracted individually from three 5 mL cultures (single colony derived) and purified using a Wizard genomic DNA purification kit (Promega, USA). After passing QC, three genomic DNA samples were pooled together, and then, genomic sequencing was carried out via PacBio SMRT sequencing technology by CD genomic (https://www.cd-genomics.com/pacbio-smrt-sequencing.html). The read length from the PacBio ranged between 2 and 20 kb. An independent NGS shot gun sequence of the genome was carried out in-house (MiSeq, Illumina, USA) and compared using GenomeMatcher to manually correct start/stop codon inconsistencies before merging into a single annotated sequence file. This complete sequence file was uploaded to the RAST (Rapid Annotation using Subsystem Technology) annotation server (https://rast.nmpdr.org/). The genome annotation and prediction of genes was performed through the Microbial Genome Annotation Pipeline by RAST [19, 20].

### Genome Mining

Analyses of the *C. tyrobutyricum* NRRL B-67062 genome for genes potentially associated with antimicrobial features were performed by searching BAGEL 3 and BAGEL 4 according to the instructions by the developers as described [21, 22] (http://bagel4.molgenrug.nl/), and Bactibase http://bactibase.hammamilab.org/main.php [23] and anti-SMASH databases for microbial secondary metabolite biosynthetic clusters ( https://antismash.secondarymetabolites.org/#!/start).

### Plasmid Construction and Overexpression in *E. coli*

PEG 446 was picked after genome mining. This protein is composted of 66 amino acids. See Supplemental Data for DNA sequences (Table S1) and protein sequences (Table S2). A similar method, as described [24], using bovine beta-casein (BBC) as a fusion protein, was employed to generate an *E. coli* codon-optimized fusion *peg*446 gene linked with BBC codons followed by the enterokinase (EK) digestion site DDDDK [24] in pET28b(+) vector. The plasmid map (Fig. S1) and fusion protein sequences (Table S3) were included in the Supplemental Data. The construct was transformed into *E. coli* BL21(DE3)pLysS (Thermo Fisher Scientific, USA) for isopropyl β-D-1 thiogalactopyranoside (IPTG)-induced expression of the recombinant protein (Fig. S2A). Western blot using HIS tag antibody was carried out to confirm the expression (Fig. S2B). The His-tagged purified fusion protein contains an EK site between BBC and PEG 446 so that PEG 446 can be released by EK digestion. The Ni column-purified BBC-PEG 446 was analyzed for antibacterial activities by Biolector (m2p-labs, Baesweiler, Germany) growth assay as described [18]. The EK-digested mixtures containing released PEG 446 were also analyzed for antibacterial activities by gel overlay assay as described [25].

### Expression of PEG 446 in Food Grade *Bacillus subtilis* WEA

A *Bacillus* expression system was adapted for secreted production of PEG 446. The *Pel* secretion signal peptide of *B. subtilis* [26] was placed after the pP43 promoter before *peg446* coding sequences in the pTTB2 vector [27] which was obtained from Mo Bi Tec (www.mobitec.com), which was sold as a Food Grade Expression system (for potential safe applications), to express and secrete PEG 446 in the media. The pTTB2-*peg446* plasmid was constructed, and the plasmid map was produced by using SnapGene software (https://www.snapgene.com/).

A single colony of *B. subtilis* WEA was used for transformation following the vendor’s instruction (www.mobitec.com). Transformed positive colonies were selected by growing on 2xYT plates containing 1% (w/v) xylose while parent WEA cannot grow on xylose-only plate due to xylose-induced activation of a toxic endoribonuclease. Transformed WEA strains containing the pTTB2-*peg44*6 and pTTB2 vector only as well as parental WEA were stored in corresponding media with 30% glycerol at − 80 °C freezer.

Single colonies from 2xYT 1% xylose plate were inoculated separately into 5 mL 2xYT with xylose for *peg446* and *pTTB2* transformed *B. subtilis*, and a single colony from 2xYT plate into 5 mL 2xYT with glucose only for *B. subtilis* WEA (parent control). All cultures were grown for 24 h, at 37 °C and 200 rpm. The supernatants of culture samples (in 1.7 mL microfuge tubes) were harvested by centrifugation (12,000 × *g*, 10 min, 4 °C) and passing through membrane (0.2 $\mu$m) filtration (CFS). About 16-$\mu$L supernatant was mixed with 4 $\mu$L 5 × SDS-PAGE sample buffer, and the mixture was heated for 5 min at 95 °C and subjected to SDS-PAGE.

For scaled up protein production, 5 mL of overnight cultures from a single colony were transferred into 1000 mL fresh medium and grown for 48 h at 200 rpm. CFS were collected by centrifugation and membrane filtration, and the final CFS were stored in − 20 °C freezer. About 190 mL CFSs (thawed on ice) were transferred to a 2-L beaker at room temperature; immediately, 420 mL saturated ammonium sulfate (4.1 M) was added slowly while the samples were stirred at 200 rpm allowing ammonium sulfate into solution. The beaker was moved to 4 °C on a stirring plate for 3 h-overnight. Samples were centrifuged in large bottles using floor centrifuge at 9000 × *g* for 2 × 30 min, and supernatants were carefully removed, and all the pellets were resuspended in 15 mL filter-sterilized 0.5 × PBS buffer pH 6.5. The samples were then dialyzed overnight against a 3500 MWC membrane at 4 °C. The dialyzed samples were checked for protein concentrations using protein assay kit (Bio-Rad, USA), adjusted to 1 $\mu$g/$\mu$L, and stored at − 20 °C.

### Antibacterial Activities by Gel Overlay

Protein samples were subjected to separation using duplicate Bio-Rad 4–20% gradient tris–glycine gels (Bio-Rad TGX) with 10$\mu$g sample and 10$\mu$L 2X laemmli buffer (Bio-Rad). One gel was used for Coomassie blue stain, and another gel was rinsed twice in double distilled water, then sliced and used for gel overlay assays against target strains *Listeria innocua* B-33088, *Limosilactobacillus fermentum* 0315-25, and *Lactococcus lactis* LM 0230.

A single colony of either *Lis. innocua* NRRL B-33088 on Brain Heart Infusion (BHI) plate (Becton Dickinson, USA) or *Lac. lactis* LM 0230 on M17 plate (Becton Dickinson, USA) [18] or *Lmb. fermentum* 0315-25 on De Man-Rogosa-Sharpe (MRS) plate (Becton Dickinson, USA) was inoculated in 5 mL of BHI broth for strain B-33088 or 5 mL M17 broth for strain LM 0230, or 5 mL MRS broth for strain 0315-25. Here, the three-letter standardized abbreviations were used to avoid confusions as recommended [28]. These cultures were grown overnight at 37 °C, 200 rpm for B-33088, or at 30 °C, 100 rpm for LM 0230, or 37 °C, 0 rpm for 0315-25. To 13 mL of corresponding soft agar (melted and cooled at 55 °C), 2 $\mu$L of the overnight culture was added, gently mixed, and poured over M17, MRS, or BHI plates respectively. The plates were incubated inside sterile biological hood at room temperature for 15 min to be solidified; then, gel slices were rinsed twice with sterile deionized water and gently placed on top of the solidified plates, and the plates were covered and incubated overnight at 30 °C for *Lac. lactis* LM 0230 and 37 °C for *Lmb. fermentum* 0315-25 and *Lis. innocua* B-33088. The plates were removed from the incubator after overnight incubation. The appearance of clearing zones was observed, and results were documented by using the Bio-Rad ChemiDoc imaging system.

### Anti Listeria Activity by Well Diffusion Assay

A single colony of *Lis. innocua* B-33088 on BHI plate was inoculated in 5 mL of Brain Heart Fusion (BHI) and grown overnight at 37 °C, 200 rpm. To 13 mL of soft agar of BHI (melted and cooled at 55 °C), 2 $\mu$L of the overnight culture of B-33088 was added, and the tube was gently inverted four times before pouring the mixture over the BHI plate. A metal replica was placed in the center of the plate and left in a biological hood at room temperature for 15 min (allowing the top agar on the plate to be solidified). Then, the replica was gently lifted, and 10 $\mu$L of cell-free supernatants from overnight cultures of corresponding *B. subtilis* parent WEA and recombinant *B. subtilis* carrying pTTB2 or recombinant *B. subtilis* carrying pTTB2-*peg446* strains were loaded into individual wells. The plate was covered and incubated overnight at 37 °C. After overnight incubation, the plate with clearing wells was taken out, and results were documented by using the Bio-Rad ChemiDoc imaging system.

### Anti Listeria Growth Assay by Microplate Reader

Additional growth inhibition analyses using *Lis. innocua* B-33088 cells as target was carried out with another inhibition assay as previously described [33] using a SpectraMax M2e Microplate Reader (Molecular Devices) with PEG 446 produced and secreted by *B. subtilis* and supernatant of the recombinant *B. subtilis* cultures were concentrated by ammonium sulfate precipitation as described above. Briefly, overnight grown cells of *Lis. innocua* were adjusted to OD600 = 0.1 by using BHI broth. Then, a 95-*μ*L cell was mixed with 5 *μ*L of PEG 446 (1.0 *µ*g/*µ*l) or control PBS buffer (pH 6.5) in 96-well microtiter plates (round bottom; Greiner) and incubated at 37 °C for 24 h, and bacterial growth was monitored by reading OD600 every 15 min. Each sample was repeated three times, and data were transferred and plotted in Excel.

## Results and Discussions

### Antibacterial Activities

**Fig. 1:**
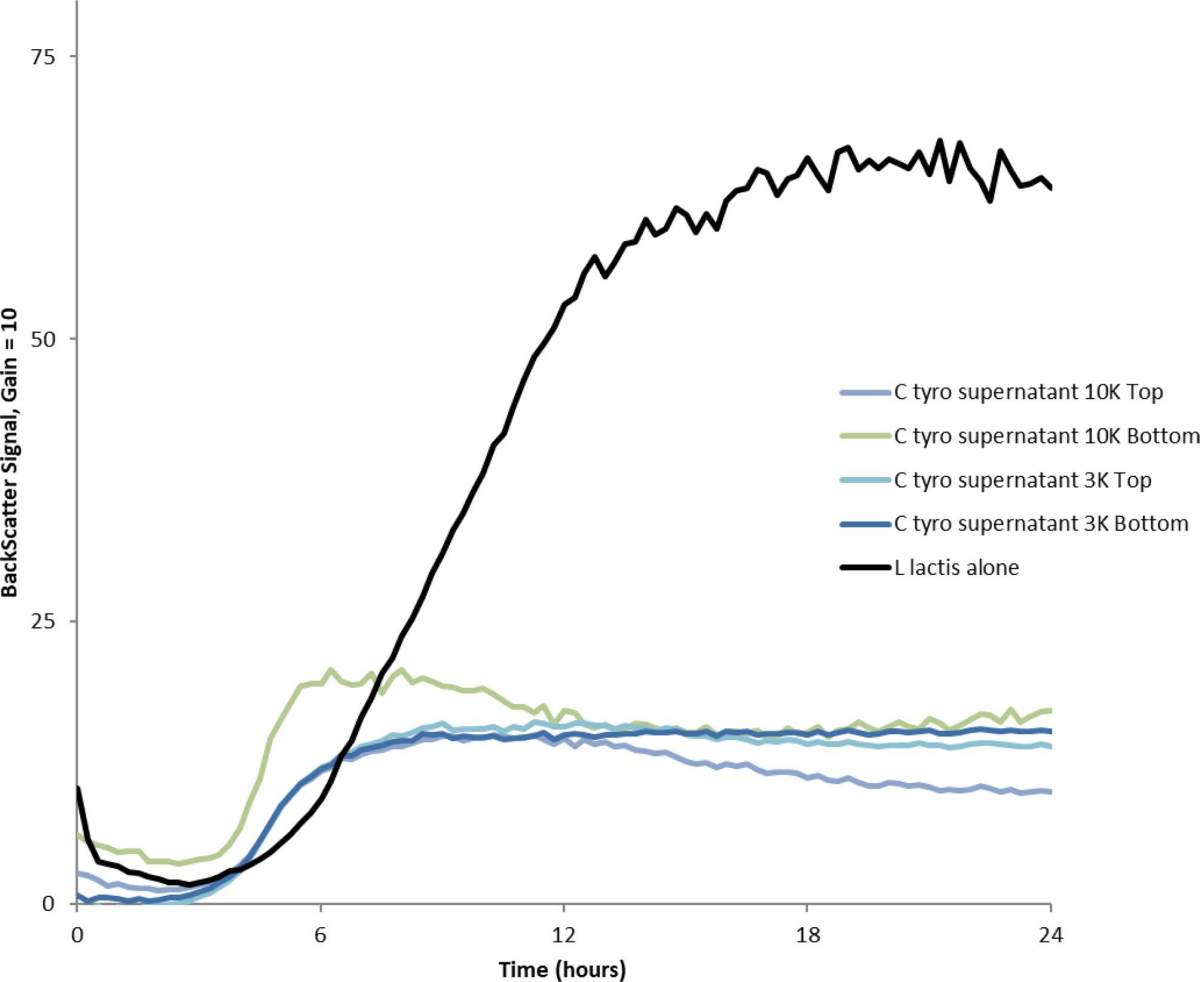
Growth inhibition of *Lac. lactis* with fractionated *C. tyrobutyricum* NRRL B-67062 cell-free supernatant (CFS). Biolector growth analysis was performed with CFS filtered with 3K or 10K MW cutoff membranes. Top-retentate, Bottom-flowthrough

Different fractions (based on size ranges after molecular weight cutoff membrane filtration of *C. tyrobutyricum* NRRL B-67062 cell-free CFS obtained from fermentation broth) were used to check bacterial growth inhibition. A total of 40 $\mu$L of each fraction was used for the Biolector screening assay against the model target *Lac. lactis* cells as previously described [18]. BackScatter signals derived from averages of triplicate assays were corrected by using both blank wells and media-only controls. Figure 1 shows that all four fractions contained antibacterial properties when compared with control replacing 40 $\mu$L CFS with plain RCM media. Since the pH of CFS was adjusted to 6.5 with 1 M NaOH, growth inhibition by organic acid seems unlikely. A separate assay was carried out later with 8.0 g of butyric acid per liter solution equivalent to the amount of butyric acid produced after overnight flask fermentation [6] was carried out without apparent growth inhibition (data is not shown).

### Genome Sequence Analyses

With the readily available sequencing technology, the whole genome sequencing of *C. tyrobutyricum* NRRL B-67062 was completed. It is noted that this genome data was obtained by PacBio and verified with MiSeq. The genome of NRRL B-67062 strain (Genbank # CP137758) has a circular chromosome of about 3.24 Mb, with an average GC content of 31.0%. There are 3114 predicted open reading frames (ORFs) and 79 functional RNA-coding regions.

*C. tyrobutyricum* genome size varies among 12 strains as reported recently. The genome size ranged from 2.99 (Cl_82) to 3.32 Mb (Cl_239), GC content from 30.49 (Cl_52) to 31.04 (Cl_117), number of genes from 2990 (Cl_188) to 3361 (Cl_239), and number of CDSs from 2883 to 3228 [17]. As mentioned earlier, whole genome analyses of different *C. tyrobutyricum* strains were reported in several laboratories. We discarded the comparison results with draft genomes and only showed data of comparing B-67062 with two complete sequences (Table 1). There are identical numbers of genes involved in TCA cycles, pyruvate metabolism, and metabolism of ketone bodies, and minor differences are seen among amino acid and vitamin B6 metabolism as shown in Table 1.

**Table 1:** Comparison of three *Clostridium* genomes by using the KEGG pathway map

KEGG map	Distinct ECs	Clostridium tyrobutyricum B-67062	Clostridium tyrobutyricum KCTC5387	Clostridium tyrobutyricum W428
Alanine, aspartate, and glutamate metabolism	43	17 (39.5%)	18 (41.9%)	18 (41.9%)
Biosynthesis of type II polyketide backbone	3	1 (33.3%)	2 (66.7%)	2 (66.7%)
Butanoate metabolism	52	11 (21.2%)	13 (25.0%)	13 (25.0%)
TCA cycle	22	7 (31.8%)	7 (31.8%)	7 (31.8%)
Glycolysis/gluconeogenesis	41	15 (36.6%)	16 (39.0%)	16 (39.0%)
Pyruvate metabolism	64	17 (26.6%)	17 (26.6%)	17 (26.6%)
Synthesis and degradation of ketone bodies	6	1 (16.7%)	1 (16.7%)	1 (16.7%)
Vitamin B6 metabolism	26	2 (7.7%)	3 (11.5%)	3 (11.5%)

**Fig. 2:**
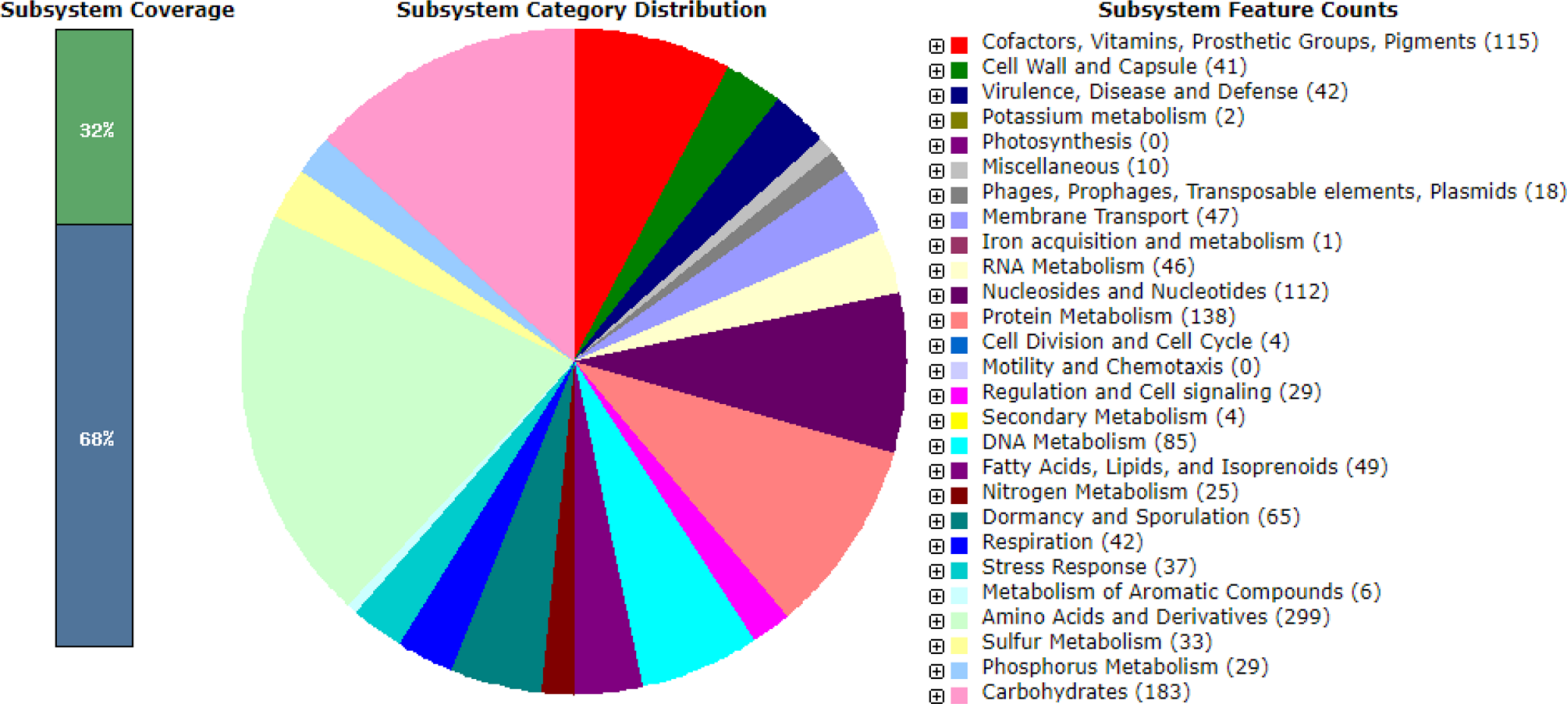
Genome analysis and functional compositions of featured subsystems for *C. tyrobutyricum* NRRL B-67062 genome. The largest subsystem appears to be amino acids and derivatives of 299 features, and the second largest subsystem is carbohydrate metabolism of 183 features and then protein metabolism (138) followed by cofactors and vitamins, prosthetic groups, and pigments (115)

The diagram in Fig. 2 shows the genome content of B-67062 and its functional compositions with featured subsystems based on RAST annotation. A RAST subsystem includes genes with closely related functions in a metabolic process, a structural complex, or a class of conserved proteins. For example, the carbohydrate metabolic pathway is grouped in one subsystem. The largest subsystem of B-67062 appears to be amino acids and derivatives containing 299 features, and the second largest subsystem is carbohydrate metabolism of 183 features. Then, protein metabolism (138) is followed by cofactors and vitamins, prosthetic groups, and pigments (115). The genome sequence analyses suggested the absence of virulence genes and antimicrobial resistance genes that were also reported in another *C. tyrobutyricum* strains [14].

### Genome Mining for Antibacterial Peptides

We examined the genome specifically for potential bacteriocin-producing genes as we detected antibacterial activities from the fermentation broth. Two clostridial bacteriocins were reported in 2003 including circularin A produced by *Clostridium beijerinckii* ATCC 25752 and closticin 574 produced by *Clostridium tyrobutyricum* ADRIAT 932 [29], but searching the genome with these two protein sequences did not reveal genes coding for similar proteins. Genome mining for bacteriocins resulted a total of 16 candidate genes (Table 2). One of the following criteria shall be met for a given gene to be regarded as a potential class II bacteriocin or related accessory gene from the database searches listed in Materials and Methods: (1) the protein should be relatively short (between 50 and 85 aa); (2) the protein might contain an N-terminal secretion signal (double-glycine or sec-dependent leader); (3) the predicted mature peptide (20 to 65 aa) can be cationic; (4) the bacteriocin-related genes involved in immunity and transport (e.g., an ABC transporter and its accessory protein) should be located immediately downstream or upstream; (5) the bacteriocin-related genes involved in regulation (e.g., a histidine protein kinase and a response regulator) should be located in the vicinity of the potential bacteriocin gene. Considering these criteria, four peptides PEG 442 (74 Aa), PEG 446 (66 Aa), PEG 912 (88 Aa), and PEG 1653 (77 Aa) were regarded as potential leads for Class IIa bacteriocin candidates, and the other 12 proteins might play important accessory roles related to the bacteriocin activities or immunity (Table 2, green highlighted the selected four). However, efforts of chemical syntheses of the four peptides were unsuccessful (data is not shown). We decided to focus on recombinant protein production of PEG 446 for the following reasons: (1) it was the smallest peptide among the chosen four and that most of Class IIa bacteriocins are smaller (between 37 and 74 Aa, Fig. S5); (2) this sequence contained the typical conserved YGKNG motif among most Class IIa bacteriocins; (3) Blast search resulted 100% matches with uncharacterized *C. tyrobutyricum* sequences (Fig. S3 WP_017751214.1 and AND85736.1) The NCBI protein entries reported that PEG 446 is a protein of unknown function. These findings suggested it would be intriguing to explore if this unknown protein was responsible for the antibacterial activity.

**Table 2:**
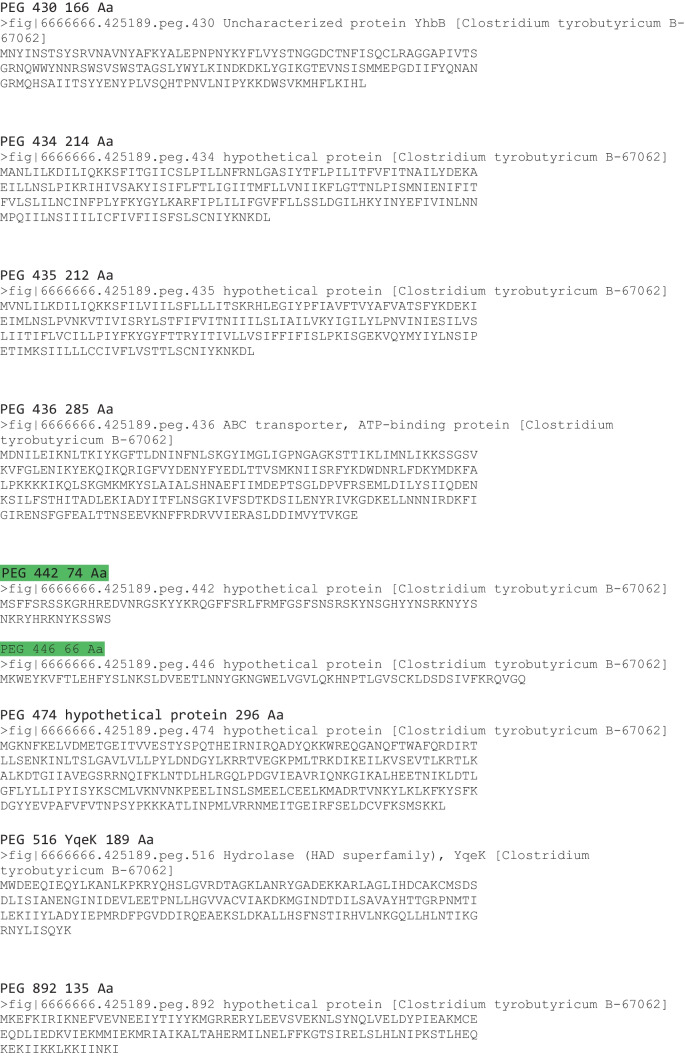

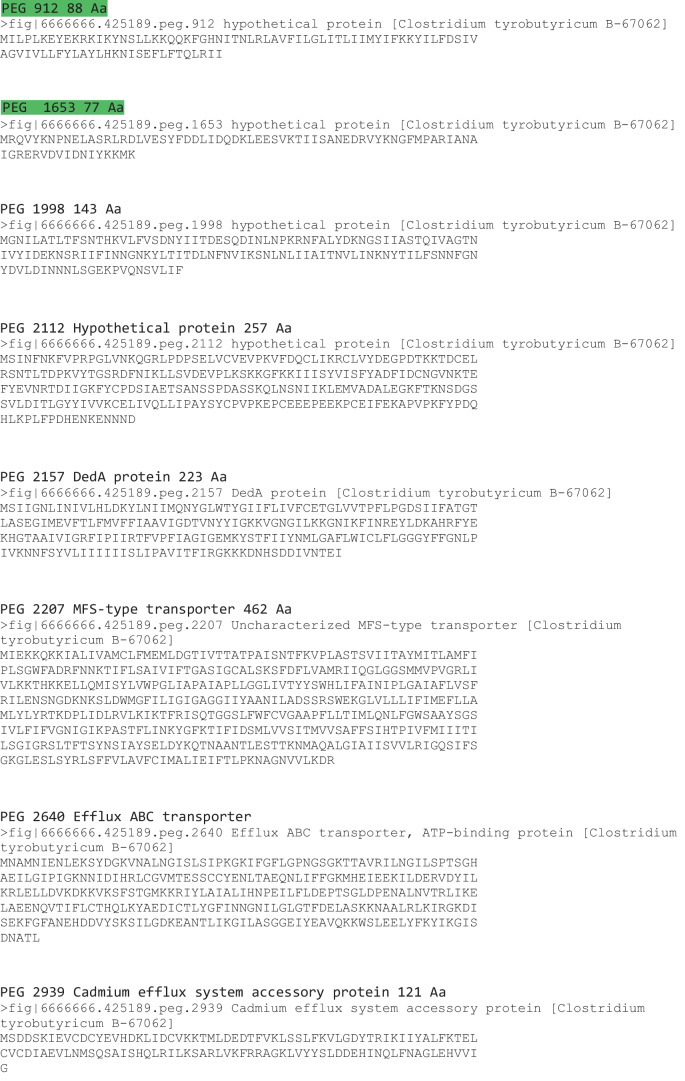
Potential bacteriocins and bacteriocin accessory proteins from *Clostridium tyrobutyricum* B-67062 genome mining

### Bioinformatics Search and PEG 446 Structure Prediction

The *peg* 446 gene (from B-67062) encodes a predicted polypeptide of 66 aa with an unknown function and was labeled as a hypothetic protein containing a conserved DUF4177 domain [30] (Fig. S3) https://blast.ncbi.nlm.nih.gov/Blast.cgi) which belongs to pfam13783 (https://www.ncbi.nlm.nih.gov/Structure/cdd/pfam13783). The DNA sequence of *peg 446* is presented in Supplemental Data S1, and the corresponding protein sequence is listed here: MKWEYKVFTLEHFYSLNKSLDVEETLNNYGKNGWELVGVLQKHNPTLGVSCKLDSDSIVFKRQVGQ (7663 Da).

**Fig. 3:**
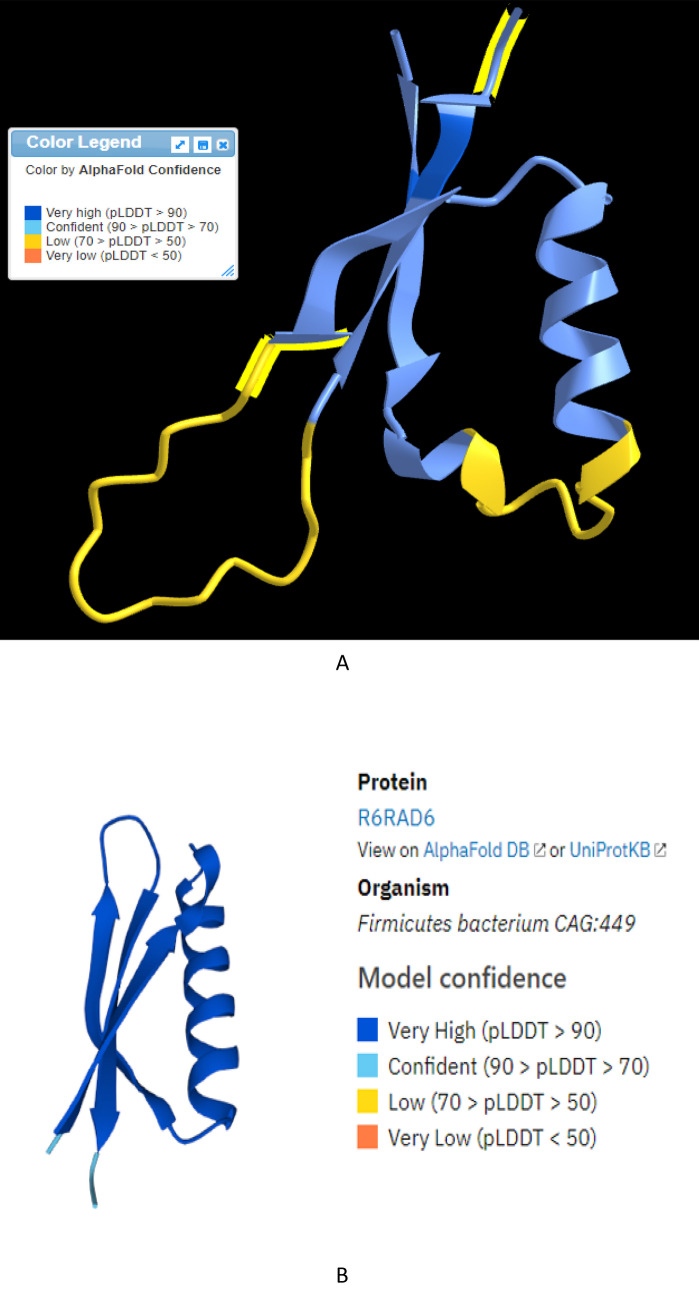
**A** Predicted protein structure model of PEG 446. This model was produced and predicted by DeepMind (18) through online bioinformatic AlphaFold analyses (https://alphafold.ebi.ac.uk/). The confidence score is between 90 and 70 (light blue) for M(MET)1 to H(HIS)12, D(ASP)21-L(LEU)40, and D(ASP)56-G(GLY)65; 70 and 50 yellow of F(PHE) 13 to L (LEU)20 and Q (GLN) 41 to S (SER) 55 and Q(GLN)66; and two amino acids E(GLU at position 4) and R (ARG at position 62) with very high confidence (dark blue). **B** R6RAD6 protein structure predicted by DeepMind with AlphaFold (18) and AlphaFold DB with higher than 90% confidence as shown in dark blue color (https://alphafold.ebi.ac.uk/entry/R6RAD6)

The PEG 446 shares 100% similarity with several unknown source microbes from recently released gut microbiome sequencing data. An additional search within the AlphaFold database found identical PEG 446 sequences (W6NBB8) from the draft genome sequence of the *C. tyrobutyricum* strain of cow’s milk isolate DIVETGP [12]. Using the amino acid sequence of PEG 446, a structure model of PEG 446 was generated as shown in Fig. 3A by DeepMind AlphaFold. It is noted that only a couple of residues in dark blue showed high confidence (pLDDT more than 90), and most structure region (including helix and sheets) of this model has pLDDT confidence score between 90 and 70 (see Fig. 3A light blue).

**Fig. 4:**
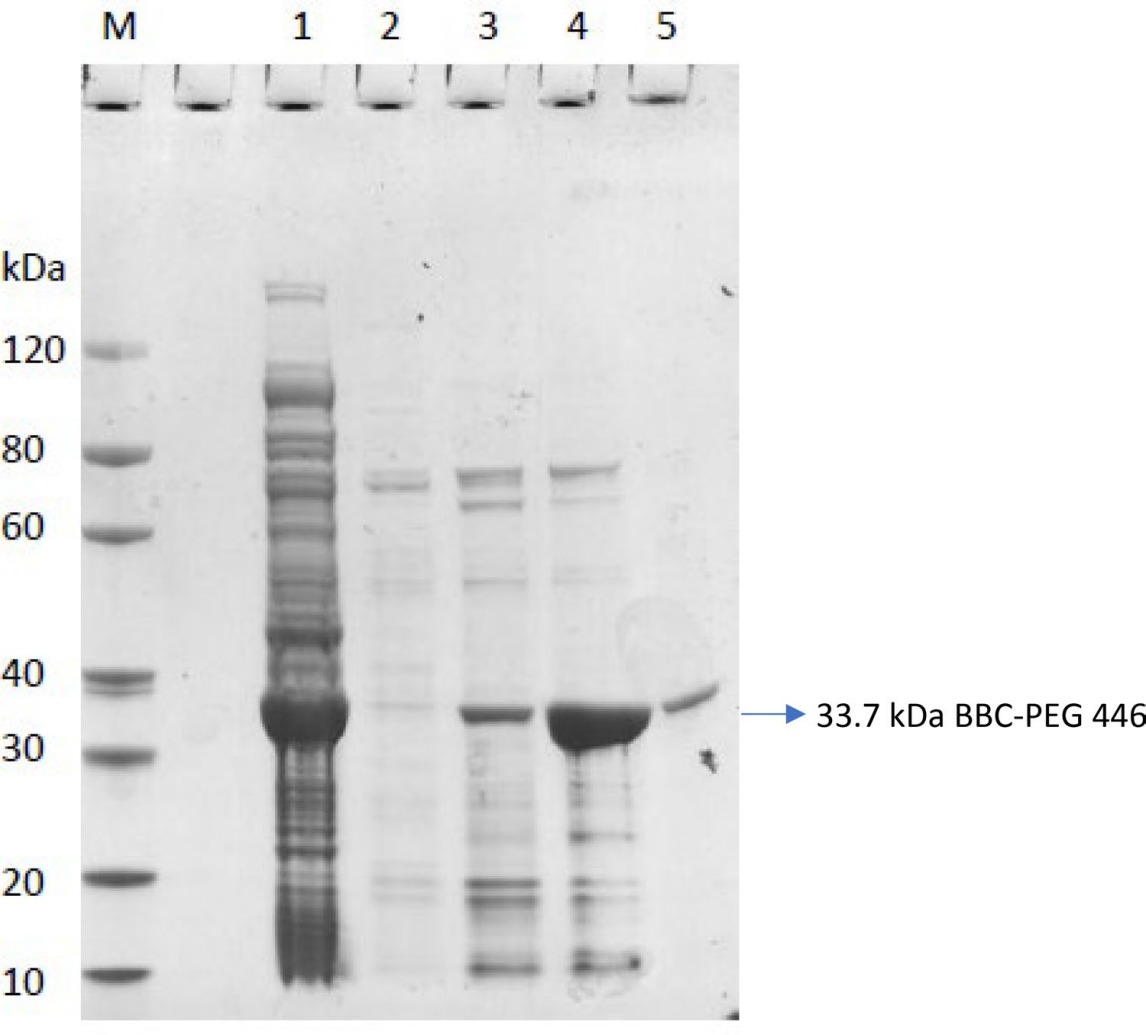
Recombinant production of *C. tyrobutyricum* B-67062 BBC-PEG 446 fusion protein (33.7 kDa) in *E. coli* BL21(DE3)pLysS cells. The overexpressed His-tagged protein was purified with Ni column; fractions were analyzed by SDS-PAGE. Lane M: protein marker; Lane 1: flowthrough; Lane 2: elution with 20 mM imidazole; Lane 3: elution with 50 mM imidazole; Lane 4: elution with 500 mM imidazole; Lane 5: Ni resin column wash

Another protein (distantly related to PEG 446) named R6RAD6 from the same DUF4177 family was identified by proteomic analyses of human gut samples. The comparison of R6RAD6 and PEG 446 resulted in 78% of the Smith-Waterman score, which exhibited 34.3% identity and 80.0% similarity in 35 aa overlaps between the two sequences. Although R6RAD6 is also uncharacterized from Firmicutes bacterium CAG:449 (https://www.uniprot.org/uniprotkb/R6RAD6/entry), the structure of this peptide was predicted with high confidence (pLDDT more than 90) by DeepMind AlphaFold (https://alphafold.ebi.ac.uk/entry/R6RAD6) as shown in Fig. 3B, and this structure model was validated independently by the Baker Lab with RoseTTAFold using the UniProt multiple sequence alignment from Pfam [31]. Since both PEG 446 and R6RAD6 belong to the same DUF4177 family, plus sequencing similarities and AlphaFold predictions (Fig. 3A, B), it is speculated that the overall structure of PEG 446 could be very close to that of R6RAD6 showing alpha-helix (residues 14–30) and three beta-sheets (parallel 1–8, 32–40, and anti-parallel 46–52). Additionally, PEG 446 homologs found in the AlphaFold database (Fig. 4SA and SB) showed similar structures.

Additional searches in the AlphaFold database of predicted structures of known Class IIa bacteriocins were carried out, and the structure models were listed (Fig. S5). It appeared that these known Class IIa bacteriocins shared similar structures including alpha-helix and three beta-sheets. One might speculate that these structure motifs are important for the antibacterial actions which have been suggested as forming holes on the cell envelope, leading to depolarization of the cell.

### Overproduction of His-Tagged PEG 446 in *E. coli*

As mentioned above, the attempt to chemically synthesize the 66 aa peptides was unsuccessful, and direct cloning and production of the native peg 446 in recombinant *E. coli* resulted no production (data is not shown). Attempts of direct expression of peg 446 in *E. coli* BL21(DE3)pLysS cells failed, likely due to the unstable structure of the smaller protein and/or potential toxicity to host cells. Recently, Class IIa bacteriocin E50-52 was successfully expressed with detectable antibacterial activities after the peptide sequences were fused with bovine beta-casein (BBC) and expressed in *E. coli* [32], so a BBC fusion PEG 446 protein was made in pET30a (Fig. S1; the exact DNA sequence was listed as supplemental data Table S2). The overexpression of the fusion plasmid in *E. coli* BL21 (DE3)pLysS resulted BBC-PEG 446 fusion protein (Supplemental data Table S3) after IPTG induction with an estimated molecular weight of 33.7 kDa, which was confirmed by using SDS gel analyses (Fig. 4) and Western blot using HIS tag antibody (Fig. S2A, B).

**Fig. 5:**
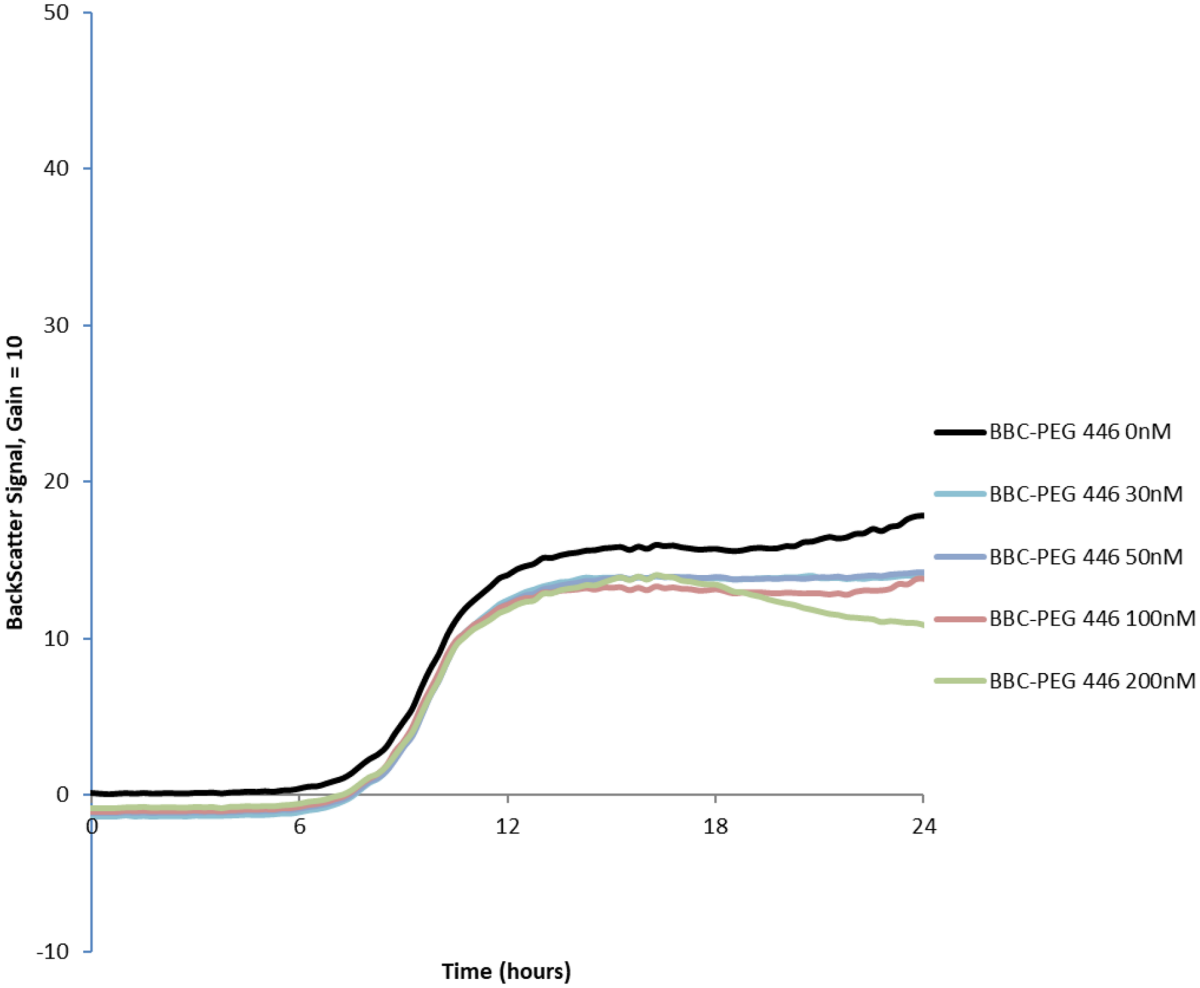
Growth inhibition of *Lac. lactis* using purified BBC-PEG 446 fusion protein (0–200 nM) produced in recombinant *E. coli*

**Fig. 6:**
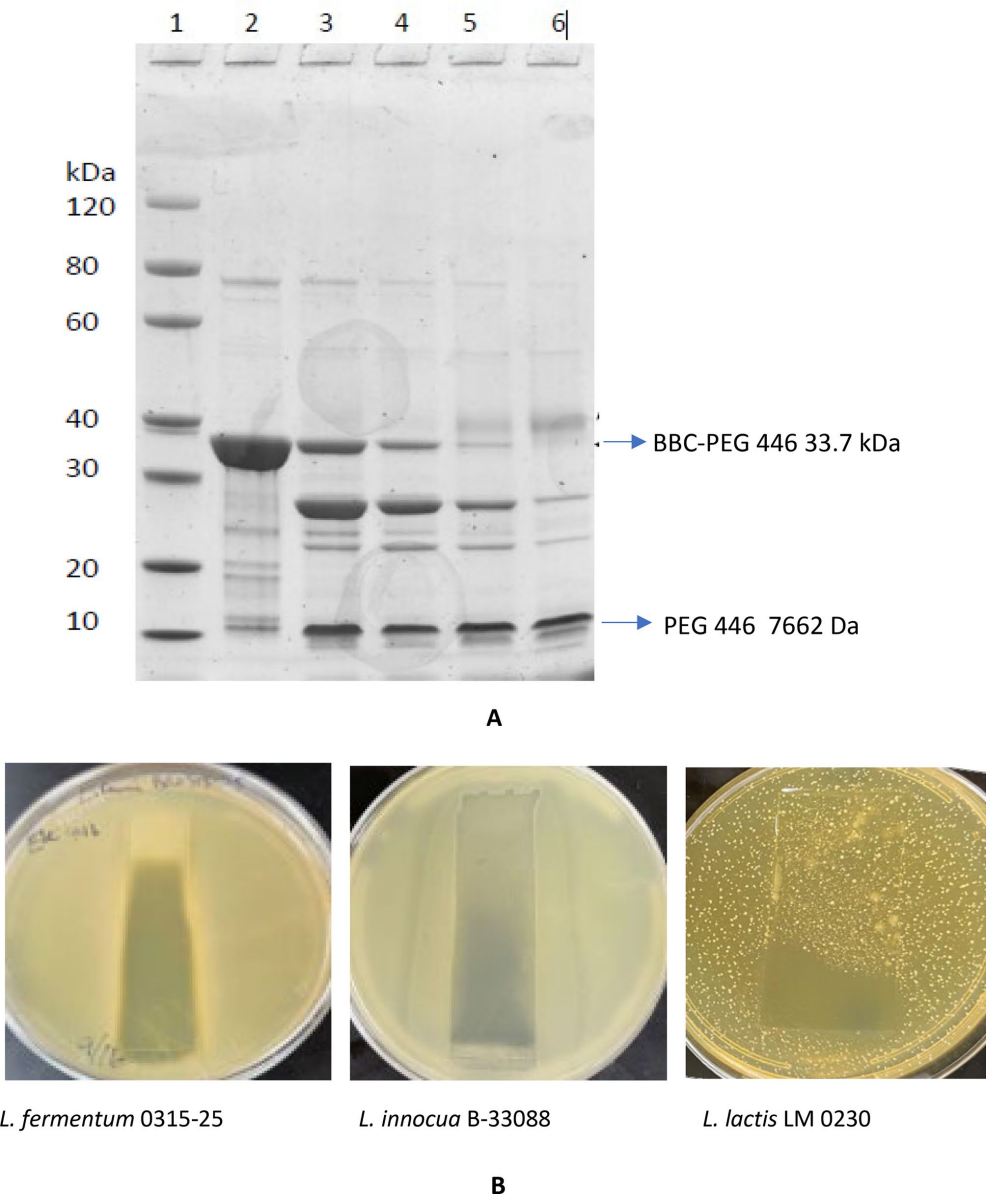
**A**, **B** EK digested BBC-PEG 446. Top panel: SDS-PAGE analyses of recombinant B-67062 BBC-PEG 446 fusion protein and EK protease digestion. Lane1: molecular weight marker (MWM); Lane 2: BBC-PEG 446 fusion protein; Lane 3: 1/100 (EK protease to BBC-PEG 446 fusion protein ratio: V/V); Lane 4: 1/50 (EK protease to BBC-PEG 446 fusion protein ratio: V/V); Lane 5: 1/20 (EK protease to BBC-PEG 446 fusion protein ratio: V/V), Lane 6: 1/10 (EK protease to BBC-PEG 446 fusion protein ratio: V/V). Bottom panel: Gel overlay assay of the EK digested BBC-PEG 446 fusion protein against three target strains *Lmb. fermentum* 0315-25 (Lane 2), *Lis. innocua* B-33088 (Lanes 3 and 4), and* Lac. lactis* LM 0230 (Lanes 5 and 6)

The purified 33. 7 kDa BBC-PEG 446 fusion protein at 0 to 200 nM concentrations in 0.5X phosphate-buffered saline was used to test its antibacterial function against target *L. lactis* cells by Biolector assays as previously described [18]. It was observed that the 200 nM dose showed better inhibition to *Lac. lactis* growth (Fig. 5). However, the inhibition appeared weaker when compared with the Biolector assay when the CFS from *C. tyrobutyricum* was used (Fig. 1). It is possible that the BBC portion of the fusion protein interferes with expected antibacterial activities. Since the fusion protein BBC-PEG 446 was linked by enterokinase (EK) digestion site DDDDK, the purified fusion protein was subjected to EK digestion with different doses of EK as illustrated on SDS-PAGE (Fig. 6A). With increased concentration of EK, the increased PEG 446 fragments were released as indicated lanes 3–6. The amount of BBC-PEG 446 fusion decreased to an undetectable level in Lane 6. To confirm activities, gel overlay was carried out after EK digestions, and antibacterial activities of PEG 446 were detected against three different target strains including *Lmb. fermentum* 0315-25, *Lis. innocua* B-33088, and *Lac. lactis* LM 0230 as shown in Fig. 6B.

It is worthy to mention the detected inhibitory activities of recombinant BBC-PEG 446 against *Lis. innocua*, which is a non-pathogenic isolate closely related to the pathogen *Listeria monocytogenes*. It was reported that the hydrophobic domain of the bacteriocin receptor mannose phosphotransferase system from *Listeria* species plays a critical role in direct interaction with the hydrophobic tip of Class IIa bacteriocin pediocin PA-1, resulting in strong inhibitory effect against *Listeria* [33]. However, no previous studies have been conducted regarding the function of PEG 446, and its antibacterial mode of function remains unknown.

It was reasoned that EK digestion can release PEG 446 from BBC-PEG 446 fusion, and a purified PEG 446 alone would be available for further studies. However, for unknown reasons, it was not possible to purify the PEG 446 protein after EK digestion of the BBC-PEG 446. Although, after the first round of the Ni column (HIS tag) purification, the purified BBC-PEG 446 was obtained and treated by EK digestion, SDS gel confirmed the release of PEG 446 fraction (Fig. 6A), but when the digested mixture was loaded on a new Ni column, assuming that BBC fragment portion (with HIS tag) would bind to the column and PEG 446 portion (without HIS tag) would come out as flowthrough, somehow, the PEG 446 fragment was also found sticked on the column and did not elute out in flowthrough fractions, and this concluded the failure to obtain a pure PEG 446 protein from recombinant overexpression in *E. coli*.

### Expression of peg 446 in *B. subtilis* WEA Strain

**Fig. 7:**
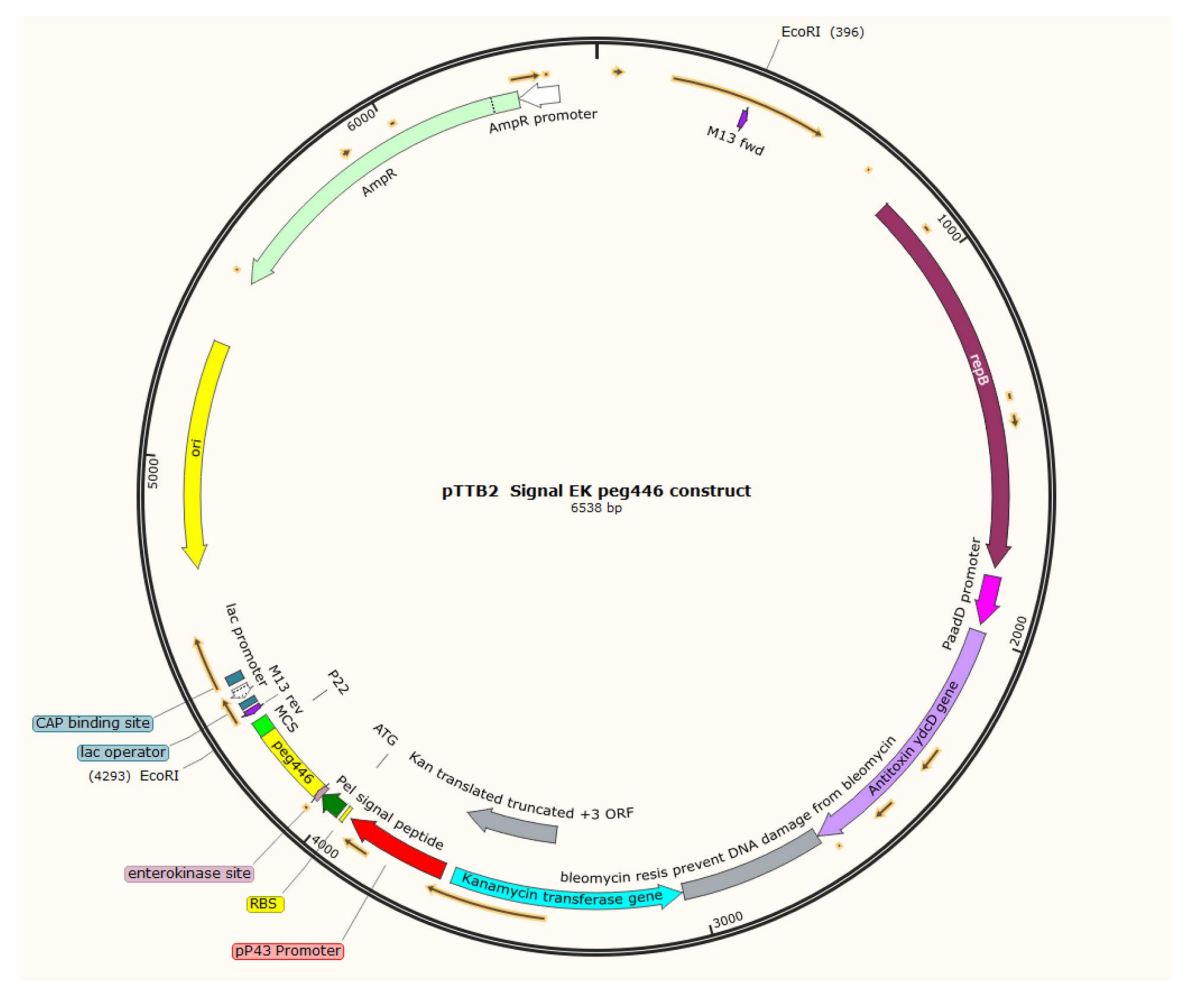
Plasmid map of pTTB2-*peg 446*. The introduction of this plasmid allows the expression of PEG 446 in *B. subtilis* that is secreted in the media by using the Pel secretory (Sec-type) signal peptide of *B. subtilis* (7). The calculated molecular weight is around 9441 Da after the cleavage of the 21 aa signal peptides (highlighted in pink below), and there is an extra 12 Aa from the vector sequences (highlighted in blue below). MKKVMLATALFLGLTPAGANADDDDKMKWEYKVFTLEHFYSLNKSLDVEETLNNYGKNGWELVGVLQKHNPTLGVSCKLDSDSIVFKRQVGQGTELARGASRCS (6538 bp plasmid)

In order to obtain secreted PEG 446 protein outside of the cell envelop and without the fusion portion, we tried the Gram-positive *B. subtilis* expression system, which has been widely used for protein expression and secretion [34]. We adapted the expression system aimed to produce only PEG 446 without the BBC fusion and added a signal peptide so that the recombinant protein can be secreted into the culture broth for fermentative production, harvesting, and purification. The pTTB2 vector was used to clone *peg 446* under control by the pP43 promoter which was linked to the pel signal peptide [26], and the resulting plasmid is presented (Fig. 7).

**Fig. 8:**
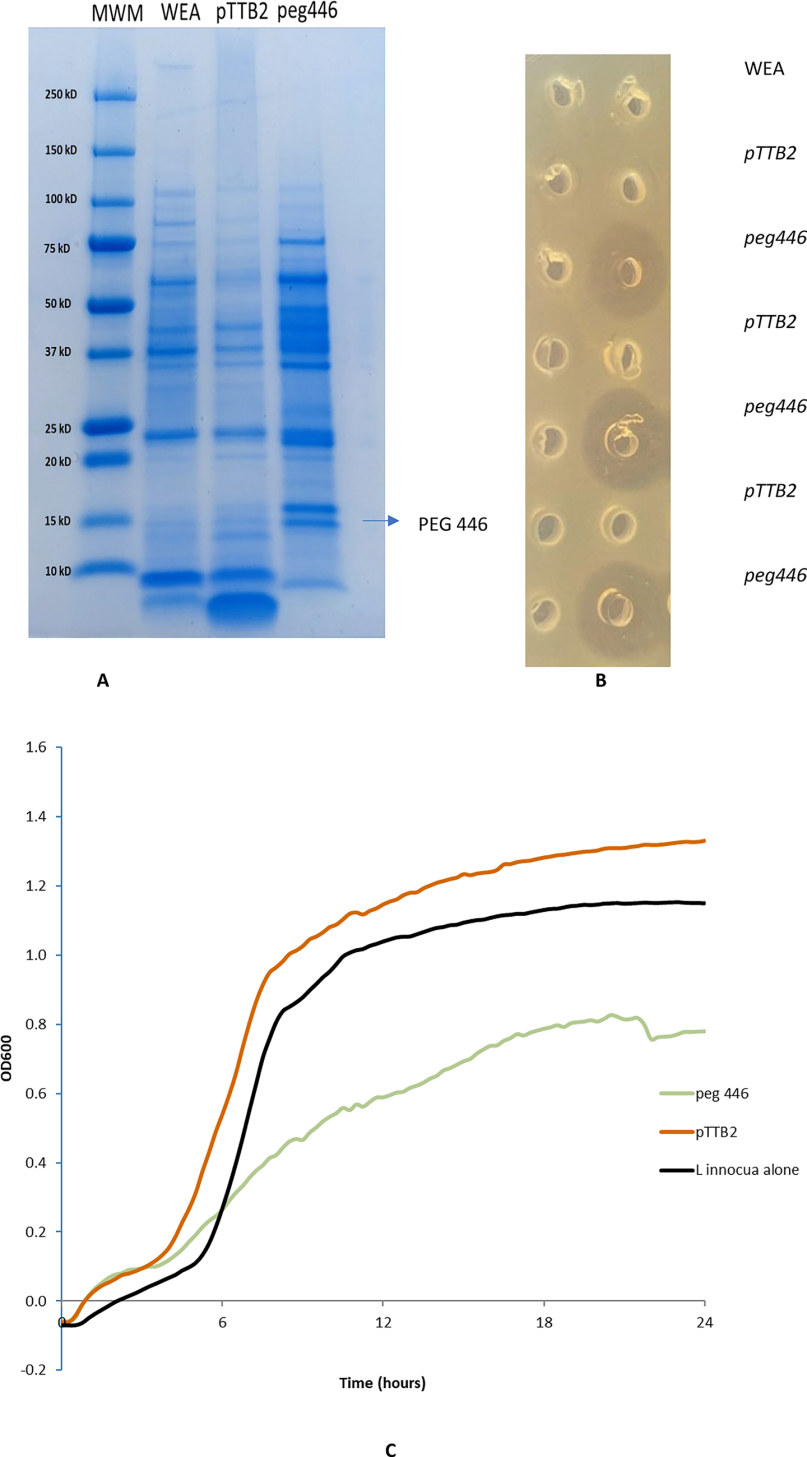
**A** SDS-PAGE stained with Coomassie blue. Lane 1: Bio-Rad All Blue Protein Std. Lane 2: WEA is protein extract from *B. subtilis* parent strain WEA. Lane 3: pTTB2 is protein extract from *B. subtilis* transformed with pTTB2 control plasmid. Lane 4: peg 446 is protein extract from *B. subtilis* WEA strain transformed with pTTB2 *peg446*. **B** Well diffusion assay against *Lis. innocua* B-33088 using culture broth after growing *B. subtilis* parent WEA, control WEA carrying *pTTB2*, and WEA expressed *peg446*. **C** Growth inhibition analyses using *Lis. innocua* B-33088 cells as target. This inhibition assay was carried out as described [35] in SpectraMax M2e Microplate Reader (Molecular Devices) with partially concentrated PEG 446. Treatment and control wells were run in triplicates, and delay in growth was calculated in percentage growth inhibition compared to control at each time point. *peg446*: protein extract from *B. subtilis* pTTB2 *peg446* recombinant strain expressing PEG 446; *pTTB2*: protein extract from *B. subtilis* recombinant strain carrying empty *pTTB2* plasmid; positive control: *Lis. innocua* cells with media only and without adding any proteins of interests

The food grade expression system uses xylose-induced antitoxin EndoB on the plasmid to neutralize the EndoA toxin produced by the WEA parent genome. As a result, the parent strain cannot grow when xylose is present in the media. A large single colony from pTTB2 *peg 446* transformants was picked and grown in 2XY supplemented with 1% xylose. The calculated molecular weight of the recombinant PEG 446 with signal peptide is approximately 11.5 kDa, and the final protein secreted outside of the cell should be about 9441 Da after the signal peptide cleavage. SDS-PAGE indicated the production of a recombinant protein, which was not present in either the parent WEA strain or the recombinant strain carrying the pTTB2 empty vector, although the protein corresponding to PEG 446 migrated slower on the gradient gel (Fig. 8A). We chose *Lis. innocua* B-33088 to demonstrate the well diffusion clearance activities since this strain is easier to grow (Fig. 8B). The results demonstrated that the cell-free culture supernatant of either *B. subtilis* parent WEA or *B. subtilis* WEA carrying pTTB2 (pTTB2) did not produce any clearance activities, while three repeated samples of cell-free culture supernatant *B. subtilis* WEA expressed *peg 446* (peg 446) resulted clearing circles of the target *Listeria* cells (Fig. 8B).

Additional growth inhibition analyses use *Lis. innocua* B-33088 cells as target was carried out with another inhibition assay as previously described [35] using a SpectraMax M2e Microplate Reader with PEG 446 produced by recombinant *B. subtilis*. As shown in Fig. 8C, the concentrated protein extract from *B. subtilis* recombinant strain expressing PEG 446 (*peg446*) reduced the growth of *Listeria* significantly while protein extract from *B. subtilis* recombinant strain carrying empty pTTB2 plasmid (pTTB2) showed no inhibitory at all, and the control is *Lis. innocua* cells with media only and without adding any proteins of interests. Although substantial amounts of secreted proteins by *B. subtilis* were removed after ammonium sulfate precipitation, the concentrated and partially purified PEG 446 is still mixed with other proteins, and the final purification has yet to be achieved. Work is in progress by using size exclusion methods.

## Conclusion

In this study, by using genome mining tools and bioinformatics analyses, we have identified, isolated, and overexpressed PEG 446 protein from *C. tyrobutyricum* strain NRRL B-67062 among several potential candidates after genome mining. The bacteriocin like PEG 446, which has not been previously characterized, is a peptide of 66 amino acids that belongs to the highly conserved DUF4177 domain (pfam13783). This is the first report of PEG 446 that shared similar structures with known bacteriocin Class IIa with a typical alpha-helix linked with three anti-paralleled beta-sheets. We have confirmed the production of recombinantly PEG 446 in both *E. coli* and *B. subtilis* and confirmed the antibacterial property of recombinant PEG 446 against Gram-positive *Lmb. fermentum* 0315-25, *Lis. innocua* B-33088, and *Lac. lactis* LM 0230 strains by using gel overlay, well diffusion, Biolector, and spectra assays. The mode of action remains to be determined with yet to be purified PEG 446.

### Supplementary Information

Below is the link to the electronic supplementary material.

Supplementary file1 (DOCX 1504 KB)

## Data Availability

All data generated and analyzed during this study are included in this published article as well as attached Supplemental Data.
