# Supplementary material for: Production of a Bacteriocin Like Protein PEG 446 from Clostridium tyrobutyricum NRRL B-67062
Source: Probiotics Antimicrob Proteins. 2024 Jan 22;16(4):1411–26. doi: 10.1007/s12602-023-10211-1 (PMC11322243; doi:10.1007/s12602-023-10211-1)
Supplement: Supplementary file 1 — Supplementary file1 (DOCX 1504 KB) [file 12602_2023_10211_MOESM1_ESM.docx]

**Production of a bacteriocin like protein PEG 446 from *Clostridium*** ***tyrobutyricum*  NRRL B-67062**

Siqing Liu^1^*, Shao-Yeh Lu^1^, Maulik Patel^2^, Nasib Qureshi^3^, Christopher Dunlap^4^, Eric Hoecker^1^ and Christopher D. Skory^1^

^1^U.S. Department of Agriculture, Agricultural Research Service, National Center for Agricultural Utilization Research, Renewable Product Technology Research Unit, Peoria, IL 61604, USA

^2^Oak Ridge Institute for Science and Education (ORISE), Oak Ridge, TN 37830, USA

^3^U.S. Department of Agriculture, Agricultural Research Service, National Center for Agricultural Utilization Research, Bioenergy Research unit, Peoria, IL 61604, USA

^4^U.S. Department of Agriculture, Agricultural Research Service, National Center for Agricultural Utilization Research, Crop Bioprotection Research Unit, Peoria, IL 61604, USA

*Corresponding author:

Siqing Liu, USDA-ARS-NCAUR, 1815 N. University St., Peoria, IL 61604-3902, USA

Siqing.Liu@usda.gov

**Running Title:** Genome mining of bacteriocins from *Clostridium tyrobutyricum* NRRL B-67062

**Keywords:** Anaerobic, Genome, Clostridia, *Bacillus subtilis*, Antibacterial, Recombinant protein

**Supplemental Data**

**Table S1** The DNA sequence of *peg 446* gene

>fig|6666666.425189.peg.446 hypothetical protein [*Clostridium tyrobutyricum* B-67062]

atgaaatgggaatataaagtatttacacttgaacacttttatagcttaaataaaagtttagatgtagaagaaacactaaataattatggaaaaaatggttgggagcttgtaggtgttttacagaaacacaatccaacgcttggagtttcctgtaaactggatagtgattcgatagtatttaagagacaagttggacaatga

**Table S2** *Bbc*-*peg 446* fusion clone DNA sequences CATATGCACCACCACCACCACCACTTAGTACCCAGGGGATCAAGAGAATTAGAGGAACTGAATGTGCCGGGCGAAATCGTGGAGAGCCTGAGCAGCAGCGAGGAAAGCATCACCCGTATTAACAAGAAAATCGAGAAGTTCCAGAGCGAGGAACAGCAACAGACCGAGGACGAACTGCAAGATAAAATTCACCCGTTCGCGCAAACCCAGAGCCTGGTGTACCCGTTTCCGGGTCCGATCCCGAACAGCCTGCCGCAGAACATTCCGCCGCTGACCCAAACCCCGGTGGTTGTGCCGCCGTTTCTGCAGCCGGAAGTGATGGGCGTGAGCAAGGTTAAAGAGGCGATGGCGCCGAAGCACAAAGAAATGCCGTTCCCGAAGTATCCGGTGGAGCCGTTTACCGAAAGCCAGAGCCTGACCCTGACCGACGTTGAAAACCTGCACCTGCCGCTGCCGCTGCTGCAAAGCTGGATGCATCAACCGCACCAACCGCTGCCGCCGACCGTGATGTTCCCGCCGCAGAGCGTTCTGAGCCTGAGCCAAAGCAAGGTGCTGCCGGTTCCGCAGAAAGCGGTGCCGTACCCGCAACGTGATATGCCGATCCAGGCGTTCCTGCTGTATCAAGAACCGGTGCTGGGTCCGGTTCGTGGTCCGTTTCCGATCATTGTTGGTCCGGGCCCGGACGATGACGATAAGATGAAATGGGAGTACAAGGTGTTCACCCTGGAACACTTTTATAGCCTGAACAAAAGCCTGGACGTTGAGGAAACCCTGAACAACTACGGCAAGAACGGCTGGGAACTGGTTGGTGTGCTGCAAAAACACAACCCGACCCTGGGCGTTAGCTGCAAACTGGATAGCGATAGCATTGTGTTCAAGCGTCAGGTTGGTCAGTAATGAAAGCTT

**Table S3** Amino acid sequences of BBC-PEG 446 fusion protein

Pink highlighted sequences are from BBC and yellow highlighted sequences are from PEG 446), note that BBC and PEG 446 was linked by enterokinase (EK) digestion site DDDDK.

HMHHHHHHLVPRGSRELEELNVPGEIVESLSSSEESITRINKKIEKFQSEEQQQTEDELQDKIHPFAQTQSLVYPFPGPIPNSLPQNIPPLTQTPVVVPPFLQPEVMGVSKVKEAMAPKHKEMPFPKYPVEPFTESQSLTLTDVENLHLPLPLLQSWMHQPHQPLPPTVMFPPQSVLSLSQSKVLPVPQKAVPYPQRDMPIQAFLLYQEPVLGPVRGPFPIIVGPGPDDDDKMKWEYKVFTLEHFYSLNKSLDVEETLNNYGKNGWELVGVLQKHNPTLGVSCKLDSDSIVFKRQVGQGTELARGASRCS

Figure S1 plasmid map of *peg 446* in pET30a
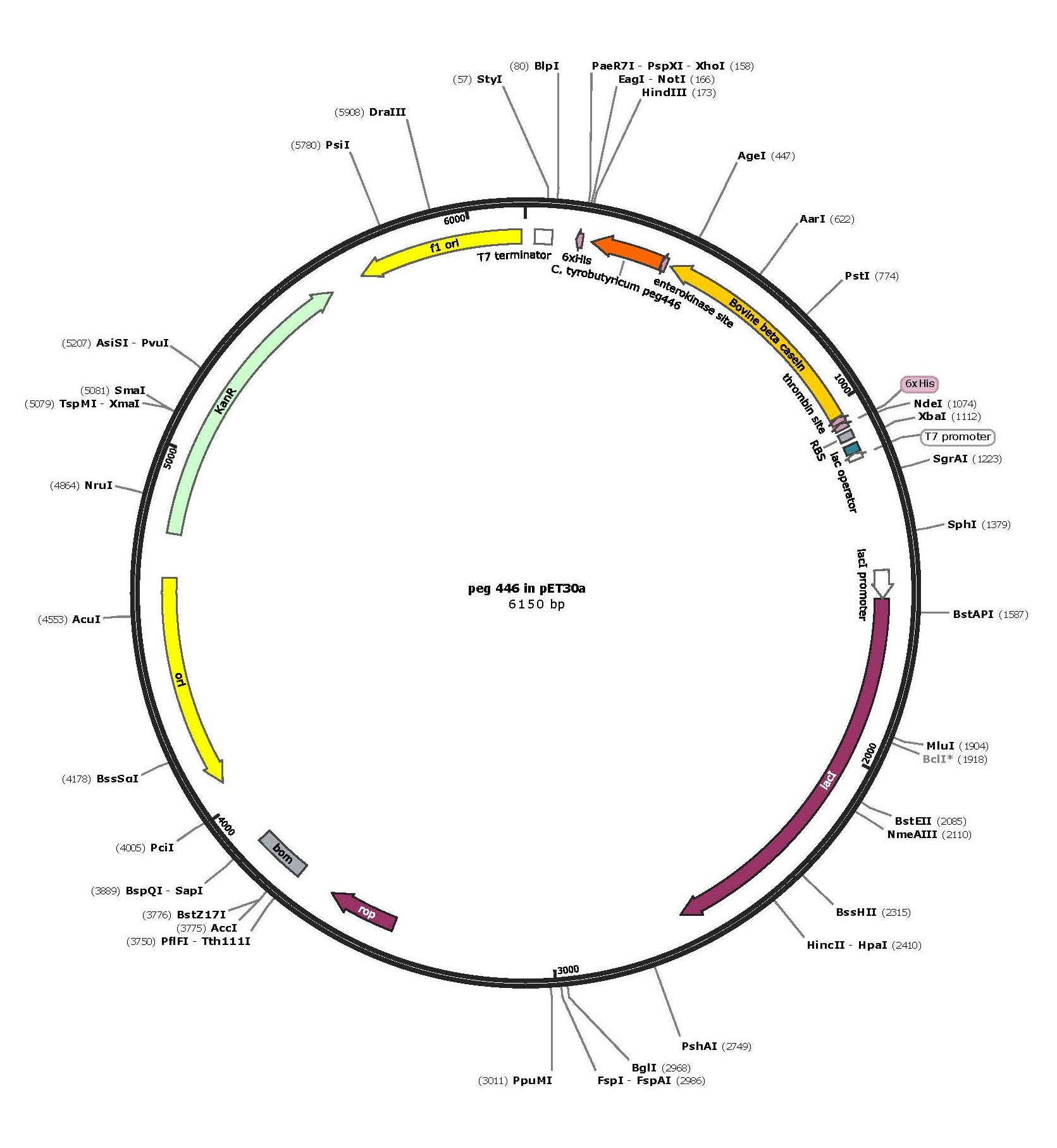


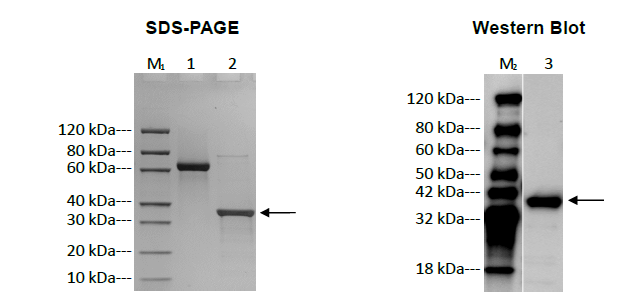


**Figure S2A** SDS-page and **Figure S2B** western blot analyses of *C. tyrobutyricum* B-67062 BBC-PEG446 purified fusion protein after IPTG induced expression in *E. coli*.

Lane M1: Protein Marker (Cat. No M00516, GenScript, USA)

Lane 1: BSA (2.00 μg)

Lane 2: Ni-column purified BBC-PEG 446 fusion protein (2.00 μg) that was over-expressed in *E. coli*.

Lane M2: Protein Marker for Western blot (Cat. No. M00521 GenScript, USA )

Lane 3: Ni-column purified BBC-PEG 446 fusion protein (0.5 μg) that was over-expressed in *E. coli* and Western blot assay using mouse-anti-His mAb (Cat. No. A00186 GenScript, USA ) as primary antibody

**Figure S3** The most recent blastx search <https://blast.ncbi.nlm.nih.gov/Blast.cgi> using *peg 446* DNA sequences (top), all are DUF4177 domain-containing proteins **Figure S3A**.

When using PEG 446 amino acid sequences and blastP search, listed here **Figure S3 B** the first 18 entries out of 100 that more than half are referred as DUF4177 domain-containing proteins.


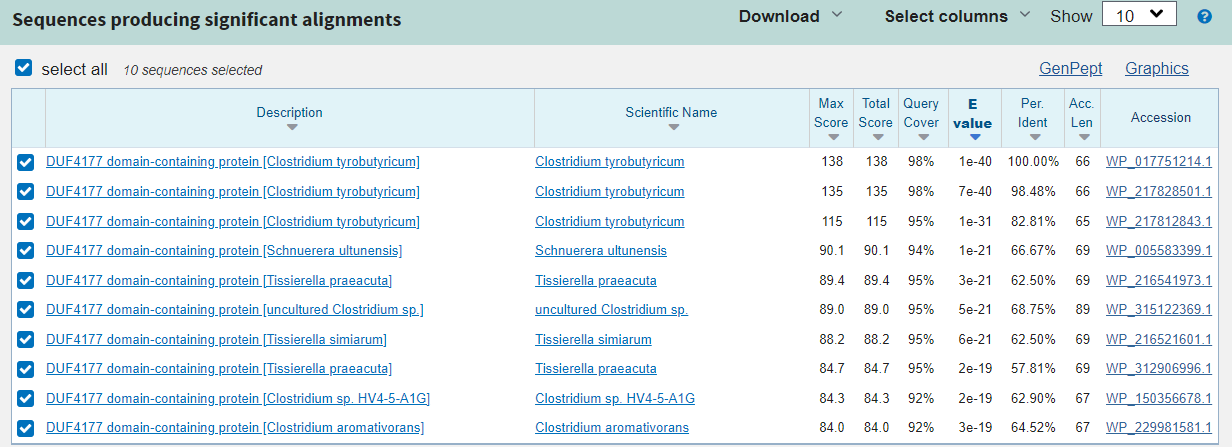


Figure S3A
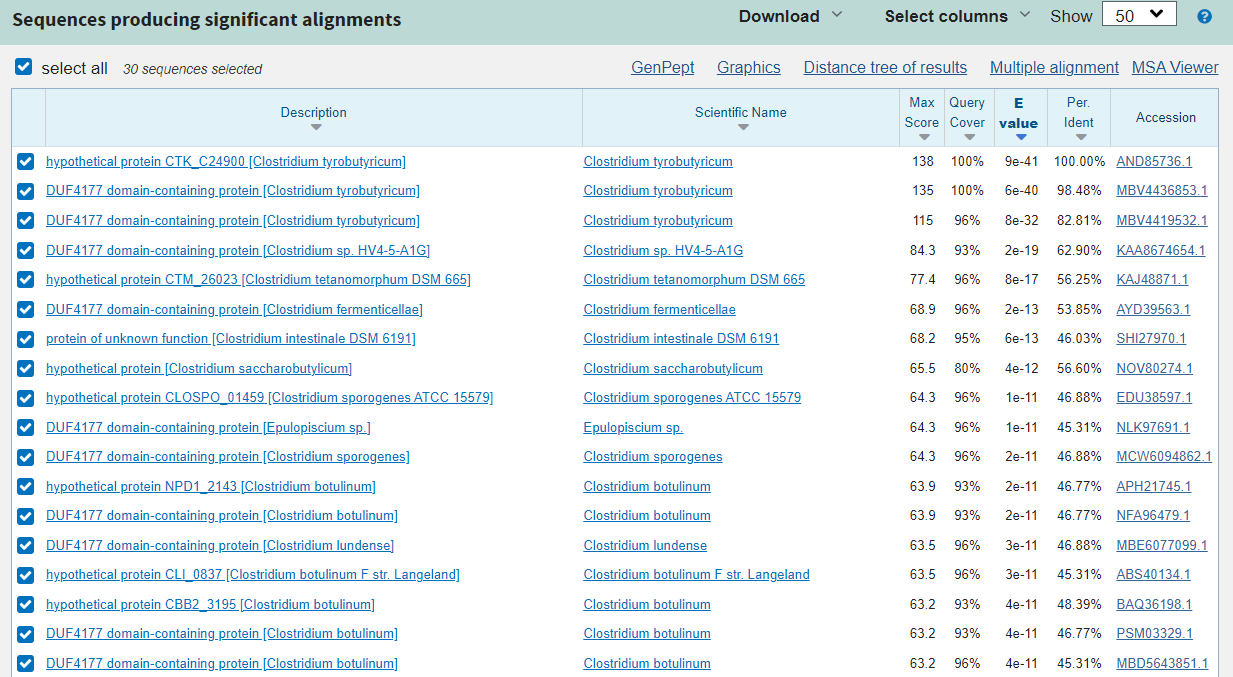


Figure S3B

**Figure S4** Similar peg 446 proteins from other *Clostridium* sequences. (https://alphafold.ebi.ac.uk)


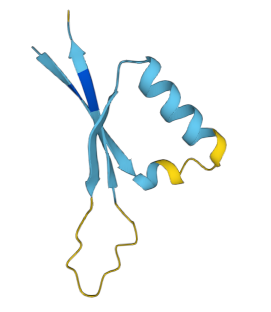


**Figure S4A** 100% identical sequences of PEG 446 with this uncharacterized protein from *Clostridium tyrobutyricum,*  currently there are two entries in AlphaFold database including W6NBB8 and A0A4P8A1J9 (66 Aa)


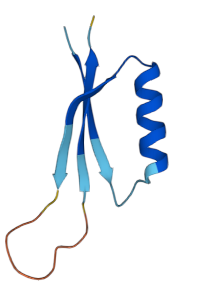


**Figure S4B** Similar DUF4177 domain-containing protein DCM59_08385 from *Clostridium* sp. A0A3C0SPR2 (64 Aa)

**Figure S5** Selected known Class IIa bacteriocin with predicted structures by AlphaFold


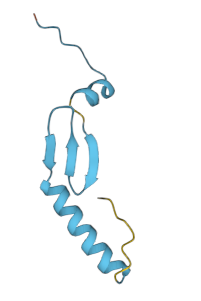

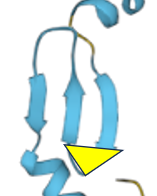


Bacteriocin pediocin PA-1 *pedA Pediococcus acidilactici* P29430 (62 Aa)

MKKIEKLTEKEMANIIGGKYYGNGVTCGKHSCSVDWGKATTCIINNGAMAWATGGHQGNHKC

The highly conserved Y-G-N-G-V/L 'pediocin box' motif is highlighted in yellow and its position Y21-V25 of the protein is indicated on the right panel as yellow triangle.


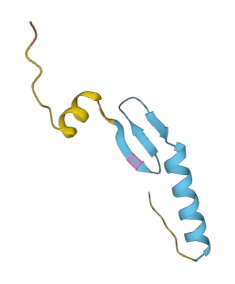


Enterocin DIW15_02340 from *Bavariicoccus seileri* A0A3D4S4Y5 (61 Aa) Red square showing the Y 22 of YGNGL motif.


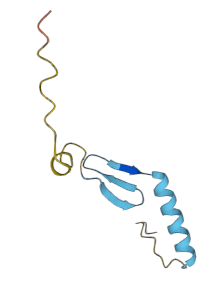


Bacteriocin piscicolin-126 *pisA* from *Streptococcus equinus* ATCC 700338 E0PG64 (72 Aa)


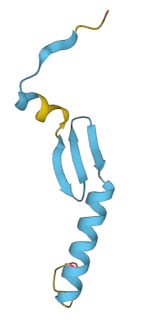


Piscicolin-126-like protein from *Carnobacterium* sp. WFPIS001 B2ZCV4 (62 Aa)


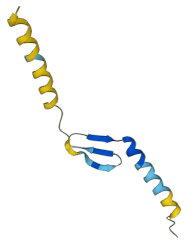


Bacteriocin BHG15_13725 from *Enterococcus hirae* A0A1V8XFG0 (71 Aa)


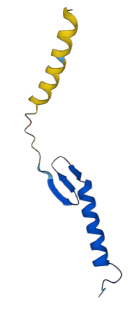


Bacteriocin hiracin-JM79 from *Enterococcus hirae* Q0Z8B6 (74 Aa)


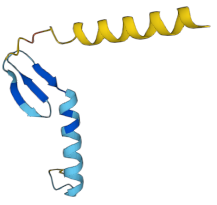


Bacteriocin hiracin-JM79 D927_02382 *Enterococcus faecalis* 02-MB-BW-10 S4C0J6 (72 Aa)


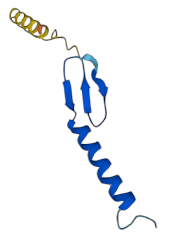


Bacteriocin A0A1W6QYG3 from *Enterococcus faecalis* (Streptococcus faecalis) (71 Aa)


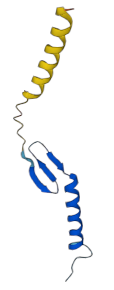


Bacteriocin *bacA* from *Enterococcus faecium* (Streptococcus faecium) Q27HG2 (74 Aa)


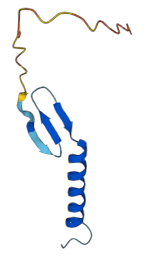


Bacteriocin *bacA* from *Enterococcus faecalis* (Streptococcus faecalis) Q47778 (67 Aa)


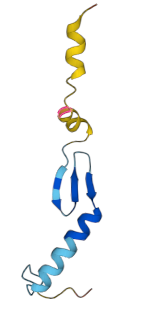


Bacteriocin from *Streptococcus equi* subsp. zooepidemicus Sz12is A0A837ELH1 (72 Aa)


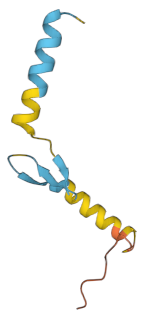


Bacteriocin ubericin-A *ubaA* from *Streptococcus uberis* A9Q0M7 (70 Aa)


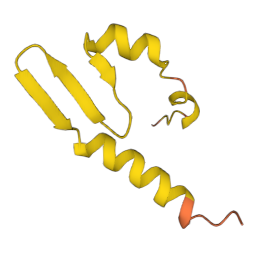


Bacteriocin mesentericin Y105 *mesY* from *Leuconostoc mesenteroides* P38577 (61 Aa)


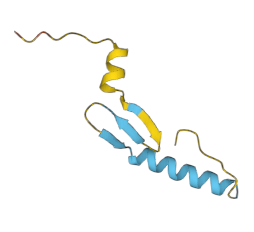


Bacteriocin pediocin PA-1 *pedA* from *Clostridium saccharobutylicum* A0A1S8NI68 (63 Aa)


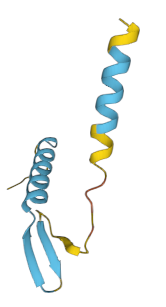


Listeriocin 743A *lisA* from *Listeria innocua* Q9AGM4 (71 Aa)


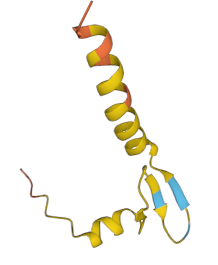


Bacteriocin sakacin-A sapA from *Latilactobacillus sakei* P0A310 (59Aa)


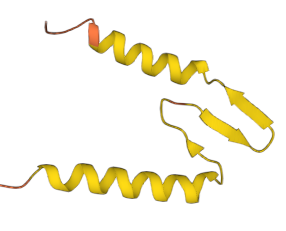


Bacteriocin leucocin-B from *Leuconostoc carnosumgo* Q53446 (61Aa)


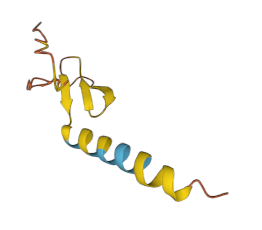


Class IIa sec-dependent bacteriocin from *Enterococcus faecium* 1,231,408 C9BUG2 (67 Aa)


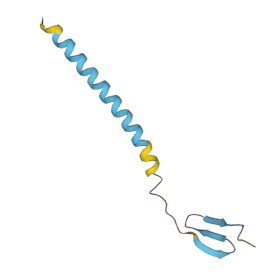


Class II bacteriocin from *Enterococcus faecium* 1,141,733 A0A7U8FPX6 (71 Aa)


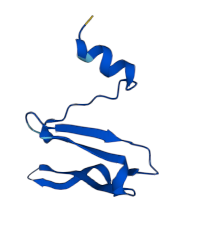


Linocin_M18 bacteriocin protein W6NKH4 (78 Aa)
